# Female and Male Perspectives on the Neolithic Transition in Europe: Clues from Ancient and Modern Genetic Data

**DOI:** 10.1371/journal.pone.0060944

**Published:** 2013-04-17

**Authors:** Rita Rasteiro, Lounès Chikhi

**Affiliations:** 1 Instituto Gulbenkian de Ciência, Oeiras, Portugal; 2 CNRS, Universite Paul Sabatier, ENFA, UMR 5174 EDB (Laboratoire Evolution et Diversité Biologique), Toulouse, France; 3 Université de Toulouse; UPS; EDB (Laboratoire Evolution et Diversité Biologique), Toulouse, France; Institut de Biologia Evolutiva - Universitat Pompeu Fabra, Spain

## Abstract

The arrival of agriculture into Europe during the Neolithic transition brought a significant shift in human lifestyle and subsistence. However, the conditions under which the spread of the new culture and technologies occurred are still debated. Similarly, the roles played by women and men during the Neolithic transition are not well understood, probably due to the fact that mitochondrial DNA (mtDNA) and Y chromosome (NRY) data are usually studied independently rather than within the same statistical framework. Here, we applied an integrative approach, using different model-based inferential techniques, to analyse published datasets from contemporary and ancient European populations. By integrating mtDNA and NRY data into the same admixture approach, we show that both males and females underwent the same admixture history and both support the demic diffusion model of Ammerman and Cavalli-Sforza. Similarly, the patterns of genetic diversity found in extant and ancient populations demonstrate that both modern and ancient mtDNA support the demic diffusion model. They also show that population structure and differential growth between farmers and hunter-gatherers are necessary to explain both types of data. However, we also found some differences between male and female markers, suggesting that the female effective population size was larger than that of the males, probably due to different demographic histories. We argue that these differences are most probably related to the various shifts in cultural practices and lifestyles that followed the Neolithic Transition, such as sedentism, the shift from polygyny to monogamy or the increase of patrilocality.

## Introduction

Major progress has been made in the use of genetic data to reconstruct the demographic history of human populations and compare alternative models of human origins [1], [2], [3]. Despite these advances, one of the most important cultural, economic and demographic revolutions in human prehistory, the Neolithic transition [4], remains the subject of continuing and hotly debated controversies [3], [5], [6], [7], [8], [9], [10], [11]. Even for Europe, where most genetic studies have been carried out, there is a major disagreement among archaeologists and anthropologists [9], [12], [13], [14], [15] and among geneticists [5], [8], [16], [17]. Some favour the hypothesis that this process resulted from an active migratory process starting in the Near East, where the domestication of Old World animals and plants began [9], whereas others believe that it was merely due to cultural contact between hunter-gathering and farming societies. These two extreme alternatives are usually encapsulated in two widely used models assuming either demic diffusion (DDM) [18] or cultural diffusion (CDM) [19]. The CDM predicts that there should be no or very little contribution in Europe from the Near Eastern populations. The genetic consequences of the DDM are much less straightforward and depend on the details of the spatial processes that took place during the expansion, including the importance of intermarriage (admixture) events between farmers and hunter-gatherers (*HG*) [1], [8], [20]. For instance, Chikhi *et al.* [8] showed that even assuming that farmers represented 90% of all the newly formed farming societies (and with only 10% of *HG*) as they expanded into Europe, the average contribution of Near Eastern genes in Europe could be as low as a few per cent, due to a dilution effect along the expansion axis, and close to zero on the western borders of Europe. They stressed a fundamental asymmetry between the two models in terms of genetic patterns and the need to use model-based approaches explicitly accounting for drift and admixture. These points were also stressed by Currat and Excoffier [1], who used more complex and sophisticated models.

Until now, one of the major limitations in the studies published is the fact that they either use mtDNA or NRY (non-recombinant region of the Y-chromosome) data, which are sometimes claimed to favour opposite models [21], even though they have never been used jointly. For instance, mtDNA data are often claimed to support CDM [5], [6], [7] whereas NRY data would support the DDM [8], [22], [23]. It is indeed very tempting to imagine that, during the Neolithic expansion in Europe, male farmers eliminated *HG* males whereas they integrated *HG* females in the newly founded farming societies, hence generating an asymmetry between male and female lineages similar to that described between Bantu speakers and African *HG* societies [24] or during the colonization of the Americas by Europeans [25].

In addition, recent technological advances have allowed the use of ancient DNA (aDNA) from early *HG* and farmer societies, hence raising new hopes that the long-lasting controversy between the CDM and DDM can be resolved. However, the recent attempts to model the colonization of Europe using ancient and modern DNA jointly [26], [27], [28], [29], have assumed very simple models that fail to incorporate crucial aspects of the demographic history of early Europeans including Neolithic farmers. They have also, in most cases, failed to use some recent advances in population genetics modelling and statistical inference. This has led to contradictory and inconsistent conclusions as we shall discuss here.

In a recent work [30], we have carried out one of the first studies where mtDNA and NRY data were analysed jointly to model ancient demographic events. Here, we continue along that road and use a simple admixture model (Figure S1) to study the spread of agriculture in Europe, by expanding the modern NRY dataset [23] and by adding modern mtDNA data [5] (see SI Material and Methods). We also take an Approximate Bayesian Computation (ABC) approach [31], [32] using one of the largest aDNA dataset available [27], to identify the demographic scenarios that could explain both modern and ancient DNA data.

We show for the first time that (i) there are no major contradictions between NRY and mtDNA data, (ii) both exhibit a clear decrease of the Neolithic contribution with geographic distance from the Near East, (iii) both favour a DDM. But there are also differences between the two markers. We show that (iv) the female effective population size was larger than that of the males, suggesting that the demographic history of males and females was significantly different before and during the Neolithic transition, probably due to differences in the migration patterns and mating systems prior to and after the arrival of agriculture. By combining evidence from both modern and ancient mtDNA we also demonstrate that (v) genetic drift and population structure were extremely important in both *HG* and farming societies, explaining why aDNA data can produce many alleles with frequencies that are significantly different from present-day frequencies and (vi) that aDNA also supports the DDM. Altogether, we propose a synthetic model of colonization that accounts for both modern and ancient mtDNA and NRY data.

## Results

### Admixture Analyses: The Neolithic Contribution Decreases with Distance from the Near East, for both NRY and mtDNA Data

**Figure 1:**
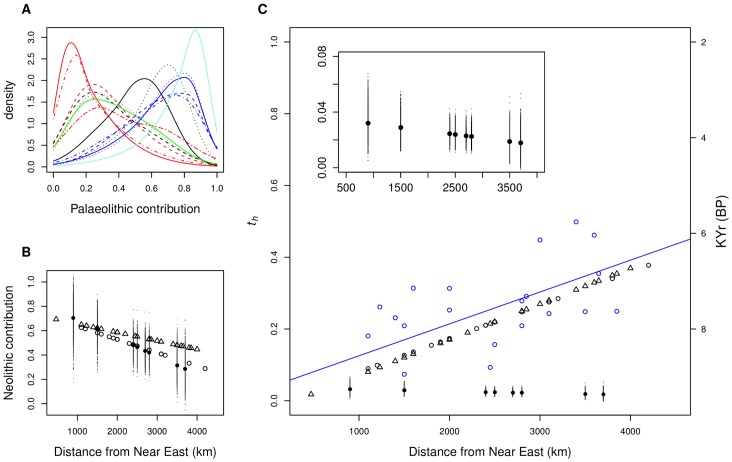
Spatial variation of admixture and drift across Europe. In (**A**) are represented the posterior distributions of the Palaeolithic contribution (*HG* contribution to modern European), for each of the populations analysed, using mtDNA data. Each curve corresponds to the analysis of a specific admixed population (Armenia − red, Caucasus – dashed red, Azeri – dotted red, Egypt – dotdash red, Iran – twodash red, Central Mediterranean − black, East Mediterranean – dashed black, West Mediterranean – dotted black, Southeast Europe – green, North and Central Europe – blue, Northeast Europe – dashed blue, Northwest Europe – dotted blue, Alps, dotdash blue and Scandinavia − aquamarine). (**B**) Linear regression of Neolithic contribution, against geographical distance from Near East. For each of the samples, one 1−*p_1_* value was randomly sampled from the corresponding posterior distribution. A linear regression was then calculated between this set of values and geographic distance. This process was repeated 1,000 times to obtain the empirical distribution of regression curves. The fitted values using mtDNA data are plotted for each of the 1,000 replicates. As fitted values are plotted, they can occur outside the range (0–1). Mean values for each population are represented by solid circles (mtDNA data) and open triangles and circles (for two different NRY datasets, Rosser et al. [23] and Semino et al. [17], respectively). In (**C**) a similar approach was used to represent the linear regression of *t_h_* (drift in the admixed populations) against geographic distance from the Near East. Mean values for each population for mtDNA and NRY datasets are plotted, with symbol codes as in (B). The close-up inset shows the mtDNA regression on a different scale for the Y-axis. Mean values for each population are represented for the sake of clarity. Calibrated radiocarbon dates of Neolithic archaeological sites [13] (see table S4) are also plotted against the distance from the Near East (blue open circles), with the linear regression represented by the blue line.

Figures 1A (mtDNA) and S2 (NRY) show the posterior distributions for *p_1_*, the Palaeolithic contribution to the European populations analysed. As expected from simulations [33], [34], the distributions are rather wide and each single population estimate has a large standard error, confirming that population genetic parameters estimated using single *locus* data are rarely very accurate. Nevertheless, when all populations are considered jointly, a clear geographic pattern is seen in both the new NRY and mtDNA (Figure 1B) datasets. This pattern shows that the proportion of Neolithic genes (1−*p_1_*) decreases from modal values of around 100% in Greece and Cyprus, to 75% in Romania, 30% in France and 20% in Spain (Figure 1B). This confirms previous results that used another independent NRY data set [8]. This trend is detected for the first time in mtDNA data, which have repeatedly been claimed to exhibit no SE-NW spatial pattern [5], [6]. Figure 1B shows that the three (two NRY and one mtDNA) datasets produce the same general trend, hence supporting a parallel decrease of female and male lineages from Neolithic farmers in the genome of modern Europeans, as we move away from the Near-East. The two NRY datasets exhibit differences, due to the fact that different populations were sampled, different numbers of SNPs were genotyped, and sample sizes were also different between the two. However, one of the NRY datasets exhibits a cline that is near identical to the cline detected for mtDNA. This strongly suggests that the difference between mtDNA and one of the NRY datasets is not greater than expected under stochasticity.

### The Neolithic Transition in the Caucasus and European islands: NRY Admixture Analyses

Another set of new results is found with the NRY samples from the Caucasus (Armenia, Georgia and Ossetia). First, the admixture level of these populations is exactly at the level expected if they had been on a SE-NW expansion axis (*i.e.* along the general direction of farmers expansion towards Europe during the Neolithic), even though they are geographically located NE of the Fertile Crescent and not NW (Figure S3A). Second, when the Caucasus data are analysed independently from the rest of the data, we find a significant geographical trend, as expected if agriculture has expanded demically from the Near East outwards in several directions, *i.e.* not just towards Europe (Figure S4A), as predicted by Renfrew’s theory linking the expansion of Indo-European with the expansion of agriculture [35], [36]. Third, the same analysis performed using populations that are unlikely to have played a major role during the Neolithic transition, due to their geographic location (*i.e.* negative controls, see SI Material and Methods) exhibit no such trend despite their much larger sample sizes (Figure S3B). Fourth, contrary to the negative controls used, several European islands population samples (East Anglia, Ireland, Cyprus and Sardinia and British Isles populations) appear to also fit within the general decrease in admixture across Europe (Figure S4B). Thus, we find clines in the Caucasus and European Islands but not in populations from the Eastern/Northern Europe.

### Drift in Paternal and Maternal Lineages: NRY and mtDNA Data Support the DDM but not the Same Demographic Histories

Genetic drift is represented by parameter *t_i_* that represents the ratio of *T*, the time since the admixture event, and *N_i_* the effective size of population *i* (see Figure S1). Thus, genetic drift in the different parental populations is represented by the parameters *t_1_* and *t_2_* for the Palaeolithic and Neolithic populations, respectively. Each of the *t_1_* and *t_2_* posterior distributions is obtained independently by the analysis of one European population (Figures S5A–B, S6A–B). First, we find that the *t_1_* posterior values are always higher than the *t_2_* values suggesting that genetic drift has been more important in the “Palaeolithic” than in the “Neolithic” parental population, in agreement with a later population size increase related with the arrival of agriculture. Second, for all the European populations analysed the *t_1_* (and *t_2_*) posterior distributions are tightly clustered, rather than spread out, even though each analysis is performed independently. Third, the different *t_1_* posterior values are more diverse (*i.e.* less clustered) than the *t_2_* distributions, which is expected if the early *HG* populations were differentiated, due to their smaller effective sizes. Fourth, the *t_1_* and *t_2_* posteriors obtained for the mtDNA datasets support much lower values than the corresponding NRY *t_1_* and *t_2_* posteriors, suggesting a much larger female (*N_f_*) than male (*N_m_*) population effective size and/or higher female gene flow.

Fifth, Figure 1C shows the results for the parameter *t_h_* which represents drift in the different European populations since the admixture event. We find that for NRY data, *t_h_* is positively correlated with distance from the Near East and with the earliest date of arrival of agriculture in the different locations based on archaeological artefacts (*i.e.* drift increases for European populations that had a *HG* lifestyle for a longer period and admixed later). In other word, the male global effective size will always be larger in the Near East (see also Figure S7). For the mtDNA data, the geographical trend is very different. Low *t_h_* values are observed in the Near East, but instead of increasing with distance they exhibit (almost) no trend (see inset in Figure 1C showing a decrease). It thus appears that the mtDNA and NRY *t_h_* results require different explanations for the demographic history of males and females, while favouring both the DDM. Sixth, differences between males and females are also observed when measures of genetic diversity (*H_e_*) and differentiation (*F_ST_*) are regressed against geographic distance from the Near East. For mtDNA, genetic differentiation between Europeans and Near Easterners increases much less with increasing geographical distance than for NRY data (Figure S8A). In agreement with this trend, differences in diversity levels are also less important in mtDNA than in NRY data (Figure S8B). Both support a higher *N_f_* and/or higher female migration rates.

### Ancient DNA, Coalescent Simulations and Model Identification Using ABC

**Figure 2:**
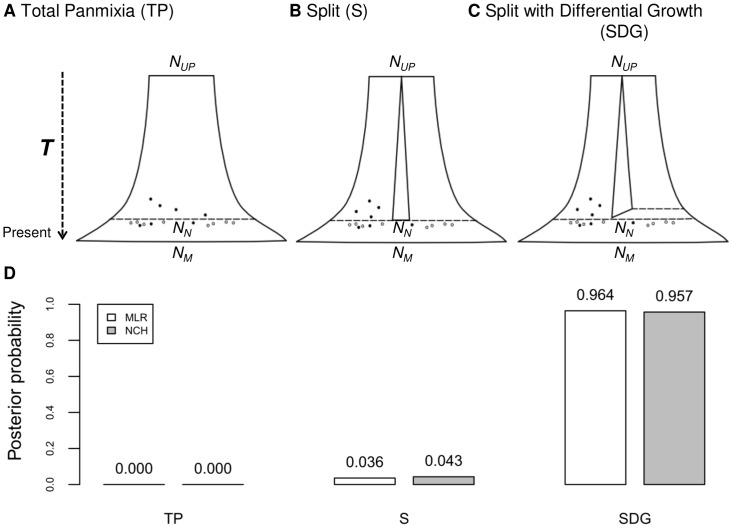
Demographic models used in the aDNA analysis and their posterior probabilities. Three different demographic models were tested using ancient and modern mtDNA data. The Total Panmixia (TP) model (**A**) follows the assumptions of Bramanti et al. [27], where *HG* and farmers were part of the same panmictic population over Central Europe and were never separated in different populations or communities. This model was used assuming a single modern female effective population size N_M_ and two periods of exponential growth: i) the first starting with an Upper Palaeolithic (UP) population of effective size N_UP_, sampled from an ancestral African female population of constant size, corresponding to the initial colonization of Central Europe 45,000 years ago and ii) the second following the Neolithic Transition 7,500 years ago, from a population of effective size N_N_. Both N_UP_ and N_N_ population sizes were allowed to vary using the same priors as in [27]. In the Split Model (S) (**B**), the UP population was structured in two sub-populations of equal size, 45,000 years ago. These sub-populations were assumed to grow independently (no gene flow), until they joined together at the beginning of the Neolithic, in Central Europe. The Split with Differential Growth (SDG) model (**C**) is similar to the S model but has a more complex splitting, in which one of the two sub-populations was allowed to have a higher growth rate between 10,000 and 7,500 years ago. In (**D**) are represented the posterior probabilities under each model, calculated using the ABC framework, for two different types of post-rejection adjustments: MLR (white bars) and NCH (grey bars).

Figure 2 represents the three demographic scenarios tested together with their posterior probabilities, using two ABC model choice algorithms on aDNA data [27]. Whether we use the multinomial logistic regression (MLR) method of Beaumont [37] or the non-linear heteroscedastic neural network (NCH) approach of Blum and François [32], the support for the Total Panmixia (TP) model is *nil*, whereas the best supported model, with a posterior probability >0.957, is the Split with Differential Growth (SDG) model which assumes a differential growth between Neolithic and Palaeolithic farmers (see also Figure S9). These results suggest that structure is required between *HG* and farmers to explain the observed data (SDG and S [Split] *vs.* TP) and that differential growth is also required (SDG *vs.* S). Furthermore, the parameters estimated for the SDG suggest that the growth rate in the *HG* populations, during the Palaeolithic, was very low or null (see table S1).

**Figure 3:**
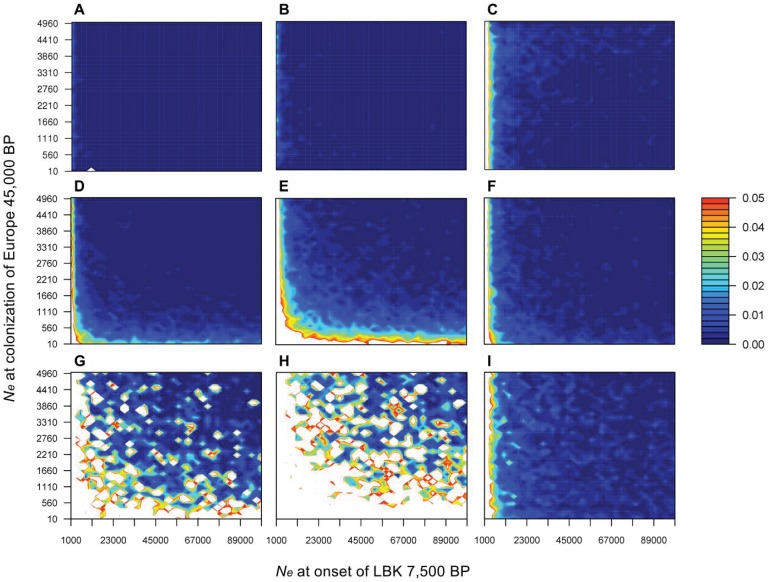
Probability of obtaining genetic differentiation (*F_ST_*) values larger than those observed in the real data. The panels in each row correspond to data simulated under the TP model (**A, B, C**), the S model (**D, E, F**) and the SDG model (**G, H, I**) (see Figure 2, for models definitions). Each column corresponds to a specific pairwise *F_ST_* comparison, namely between *HG* and early farmers (**A, D, G**), *HG* and modern Europeans (**B, E, H**), and early farmers and modern Europeans (**C, F, I**). The x- and y-axis represent the values used for the female effective size N_N_ (at the onset of the Central European Neolithic 7,500 years ago) and N_UP_ (45,000 years ago), respectively. The colour key gives the probability of obtaining a *F_ST_* value equal or greater than that observed. The white shaded area corresponds to parameter combinations for which this probability is greater than 0.05.

The same kind of results, but using another approach, is shown in Figure 3. This figure represents the estimated probability of obtaining *F_ST_* values that are equal or higher than those observed in the real data (P_S>O_), for the three scenarios. A two-tailed test was also applied and the results were (qualitatively) identical i.e. the result of the statistical test did not change (not shown). The data simulated under the TP model (Figure 3A–C) show results identical to those obtained by Bramanti and colleagues [27], hence validating our simulation approach and the exaggerated simplicity of the model used by these authors. For this model, the parameter space explaining the observed data is extremely limited. However, as soon as structure is incorporated in the models (S and SDG), the number of parameter combinations (N_UP_ and N_N_) for which large *F_ST_* values are observed becomes very large hence allowing for many realistic scenarios to explain the observed data. This is true for the S model (Figure 3D–F) and even more when we introduce differential growth in the model (Figure 3G–I). For instance, the P_S>O_ values in the SDG model panels can be as high as 0.99 for the *HG vs.* farmer comparisons or as high as one for the *HG vs.* modern European comparison, showing that simple structured models produce high *F_ST_* values for reasonable parameter values. Conversely, the simulations for the TP model have maximum P_S>O_ values of 0.018 for the first comparison and 0.032 for the latter, in agreement with the values found by Bramanti and colleagues [27] (see table S2).

## Discussion

### Both Contemporary NRY and mtDNA Data Support DDM, but Tell Different Demographic Histories

Our analyses, using contemporary data, suggest that there is a parallel decrease in the NRY and mtDNA Neolithic contributions to the European populations with increasing distance from the Near East. This is not compatible with a model of cultural diffusion and requires demic movement of both male and female farmers, from the Near East, as agriculture spread into Europe, in agreement with archaeological data [12], [13], [15], [38]. This parallel decrease also suggests that both males and females admixed with the local Palaeolithic populations that inhabited Europe at the time, resulting in a progressive dilution of the Near Eastern genes. We also found that the demic diffusion process was centrifugal, with samples from the Caucasus fitting in the general trend, as was already suggested by Renfrew [35] and others [39] and in agreement with linguistic data too [36]. Moreover, the European islands appear also to fit within this trend. This suggests that the sea did not represent a major barrier to the Neolithic expansion and that the peopling of these islands was not subjected to major drift effects or radically different admixture histories compared to neighbouring continental populations [12].

It therefore appears that, when we use one coherent statistical framework, both datasets from male [8], [23] and female [5] markers, support the DDM. These results are at odds with the original conclusions drawn by Richards *et al.* [5] (*i.e.* using only mtDNA), who advocated that mtDNA data favoured the CDM. However, they are in agreement with the clines described by Rosser *et al.* [23] (*i.e.* only with NRY data). It is worth noting that the methods used by the two studies are not comparable. Richards *et al.* [5] used the age of mtDNA mutations and haplogroups to date major demographic events. This kind of approach has been criticised as it can lead to misinterpretation of the data [3], [10], [40]. Rosser *et al.* [23] used spatial autocorrelation methods instead, to identify statistically significant clines. This method has been similarly criticised, as a cline in itself does not indicate the time at which it was established. Model-based approaches, like those applied here, explicitly state the assumptions used to make inference and are probably the most suitable to infer demographic parameters [1], [8], [41], such as the Neolithic contribution to European populations.

The fact that extant NRY and mtDNA both support the DDM does not imply that other details of the male and female demography were identical, particularly in relation with the amount of drift experienced by each sex [42]. Indeed, our results point to a higher *N_f_* over *N_m_*, in agreement with the larger coalescence times for mtDNA [43], [44]. But before addressing this issue and proposing a model accounting for these results we turn to the aDNA results.

### aDNA Supports Demic Diffusion

The first aDNA study using model-based approaches, on samples identified as Linear Pottery Culture (LBK), argued in favour of CDM [26]. Later, the same LBK data was compared to samples from Palaeolithic/Mesolithic archaeological sites and modern data from the same region, by Bramanti *et al.* [27]. They interpreted the genetic differentiation observed in the real data as being too high to “*be explained by population continuity alone*” [27], hence arguing for a Neolithic immigration in Central Europe. These two studies [26], [27] had in common that all DNA samples, ancient and modern alike, were assumed to belong to the same panmictic population (see Figure 2A). While this may seem surprising, the model assumed in these two studies is the one that we call Total Panmixia. This model assumes that there was no population structure and that *HG* and farmers were allowed to mate freely, making the distinction between HG and farmers unclear, to say the least.

What our new aDNA simulation framework suggests is that it is actually possible to explain the large genetic differentiation between samples if we explicitly model both population structure and different population growth rates between Neolithic and Palaeolithic populations before they admixed. In a recent work, Haak and colleagues [29] also allowed for some population structure, namely between populations of Central Europe and the Near East. Their results suggested an affinity between the first LBK farmers and modern Near Easterners, but they still could not explain the high population differentiation encountered between the LBK farmers and present-day Central European populations. On the contrary, our SDG model, could explain the high *F_ST_* values encountered between *HG* and farmers and between farmers (or *HG*) and modern-day Central Europeans. We believe that the main difference with the Haak *et al.* study [29] is that they did not allow variable population growth rates in their simulations. However, by varying the growth rates between *HG* and farmers, as between the onset of farming and the following period, we could explain these high *F_ST_* values.

Differential growth between farmers and *HG* is supported by anthropological and archaeological data [45], [46]. Indeed, at the onset of the Neolithic expansion in the Near East and in the front of the wave of expansion, it has been shown that a very high growth rate is expected from the colonizing populations until their size reaches the new carrying capacity ceilings [45]. Interestingly, our estimates suggest that the female growth rate remained quasi-constant during the Palaeolithic, and that there was an expansion with the advent of farming, which is also in agreement with archaeological data [47], [48]. Such an increase in *N_f_* could also be explained by an increase in gene flow following the arrival of farming, for instance if it was accompanied by a change in post-marital residence patterns in females. This is in agreement with a simulation study by Rasteiro et al. [30]. These authors simulated genetic data (mtDNA and Y-chromosome) for 45 scenarios by varying the amount of admixture between HG and farmers populations and the patterns of post-marital residence behaviour, hence allowing for a shift after the arrival of agriculture. This is also in agreement with strontium data recently published demonstrating a sudden increase in female gene flow after the arrival of agriculture in the Balkans [38] or in the LBK [49].

### Towards an Integrated Model of Neolithic Transition

Altogether, the work presented here allows us to draw a coherent integrated model for the Neolithic transition in Europe which accounts for both the congruent admixture results between mtDNA and NRY data, their difference in terms of diversity and differentiation (drift), and the constraints imposed by the aDNA data. On that basis, we propose (i) an establishment of farming communities in Europe by a demic diffusion process, with an origin in the Near East, in agreement with archaeological [12], [13], [15], [46], [50] and anthropological studies [14], [51], [52], along with a process of admixture with the local *HG* [51]; (ii) a spread in different directions from the Near East, with the Caucasus and European Islands being part of this gradual expansion, in agreement with Renfrew’s theory of Indo-European languages [35], [36]. Furthermore, we propose that (iii) both male and female farmers were involved in this demic movement in agreement with strontium data [38], [49], and that (iv) the demographic histories of the two sexes were probably different during and perhaps before the Neolithic transition. In particular, we propose that the difference in the amount of drift experienced by males and females can be explained by a change in the patterns of gene flow and by a shift in human mating systems, from polygyny to monogamy during to the Neolithic transition. Below we go through the rationale and data that corroborate this scenario.

As noted above, one of our main results is that *N_f_*>*N_m_* and/or that migration rates were higher in females compared with males (Figure 1C). Anthropological, linguistic and archaeological evidence suggest that the transition from hunting-gathering to farming or herding communities usually leads to an increase in patrilocality (*i.e.* when the marital residence is the groom’s birthplace) due to the fact that males tend to control and inherit wealth (*i.e.* the land or the herds), hence leading to higher female migration rates [49], [53], [54], [55], [56], [57], [58], [59]. Given that forager communities do not accumulate wealth, migration patterns are more likely to be symmetrical, and this is indeed what has been observed. In other words, sedentism that accompanied the Neolithic transition [60] is expected to have led to a decrease in male gene flow, whereas female gene flow would either have remained constant or would have increased to compensate the decrease in male gene flow. This would explain two of our results, namely the higher mtDNA diversity, the higher NRY differentiation, and the higher difficulty found by several authors to identify clines in mtDNA data, compared to NRY. Interestingly, this would also be in agreement with the larger coalescent times described for mtDNA compared to NRY [43], [44] and would partly explain the results and interpretation of Richard et al. [5].

Another cultural change that is thought to have taken place in Europe during the Neolithic transition is a shift from polygyny to monogamy [61], [62]. In fact, several Neolithic burials [55], [58] show evidence of nuclear families, which may reflect a monogamous marriage system. A shift from polygyny to monogamy would have the effect of decreasing male variance in reproductive success, since more males would now be able to mate, and consequently would increase *N_m_*. This could result in a signal of population growth in NRY data that would be more recent compared to that observed in mtDNA and is exactly what Dupanloup and colleagues [63] have argued and found. Our results are in good agreement with theirs. Indeed, we found that *t_h_* increased in males but not in females as we moved away from the Near East (Figure 1C), with *t_h_* being the ratio of *T*, the time since the admixture event, and *N_h_*, the effective size of the admixed population. Given that *T* necessarily decreases as we move away from the Near East, an increase of this ratio suggests that the decrease of *T* was compensated by a rapid increase in *N_h_*. In other words, the admixture process between HG and farmers led to a very rapid increase in the effective size of the male population whereas this increase was more limited in females. Indeed, a shift from polygyny to monogamy would have less influence on *N_f_*, which would anyway be higher than that of males, due to their lower variance in reproductive success. Altogether, a model in which human societies began to adopt farming as a means of subsistence, with the correlated patrilocality and monogamy as a mating system, would be in agreement with all the results presented here, including the aDNA (for instance it was rather impressive to find that the most probable scenarios, independently inferred no significant growth in Palaeolithic females). It also allows us to put in a single picture, results from several genetic and anthropological studies.

While we claim that a more coherent picture emerges from our results, we cannot claim that other scenarios could not also explain the results. Many layers of complexities could be added. For instance, female hypergamy (*i.e.* the fact that lower social status women are more likely to mate with males from a higher status than the opposite) has been described in several human migration and colonization events [24], [25], [64], and it is believed that it probably happened during the Neolithic transition in Europe [51], with *HG* females marrying into farmer communities [65]. Qualitatively, female hypergamy would increase female mobility and lead to low levels of mtDNA genetic differentiation between populations. Thus, one should expect lower mtDNA gradients and (almost) no geographic trend in drift, which is exactly what we see. The exclusion of HG males would lead to an increase of NRY genetic differentiation, explaining the clear geographic trend found in genetic drift. However, we must add that this scenario, which may indeed have taken place, would not as easily fit with the admixture patterns that we find and which are similar in males and females Also, it does not fit with the recent strontium isotope data [38], [49]. Thus, at this stage, we would be cautious before arguing for or against female hypergamy. We also insist on the fact that the patterns identified here correspond to global patterns, and are not in contradiction with regional studies arguing against the demic diffusion. Several processes are likely to have taken place during the millennia corresponding to the arrival of farming communities in Europe. Similarly, it is increasingly clear that different routes (coastal or continental) were followed by different groups of humans. Still, the genetic data point to a major input from Near Eastern populations. This cannot be explained by cultural diffusion at a European scale and, as we have argued repeatedly, the general approach using the age of haplogroups or haplotypes to reconstruct human prehistory still awaits formal validation [3], [8], [11], [66], despite the large literature that uses it [10], [11], [40].

Our study represents the first attempt to integrate contemporary mtDNA and NRY data, together with aDNA. This has allowed us to draw a coherent picture of the Neolithic Transition in Europe, which not only provides an explanation for the patterns of genetic diversity found today and in our past, but also for the apparent contradiction between phylogeographic and model-based studies. The aDNA modelling approach described here could be applied to other aDNA datasets and we have applied it to data from an Iberian Neolithic population [67] The results from these independent data appear to validate the suggestion that structured models with varying growth rates explain better the genetic distances observed between ancient and modern DNA than simpler models. The Neolithic transition in Europe is one of the most studied periods of human prehistory and the source of much debate. It is our hope that the work presented here may help provide a consistent framework to address certain aspects of this long-standing controversy.

## Materials and Methods

### Estimating Admixture/Interbreeding between Palaeolithic HG and Neolithic Farmers Using Extant Genetic Data

We applied a Bayesian full-likelihood method based on a simple admixture model that assumes that in a given moment in the past, an “admixed” population *H* (representing the European populations), is formed by members of two independent parental populations, *P_1_* and *P_2_* (representing *HG* and the farmers, respectively), whose contributions to *H* are *p_1_* and *p_2_* (*p_2_ = 1−p_1_*), respectively (see Figure S1). After the admixture event, the three populations are isolated and assumed to evolve independently under pure genetic drift, represented by parameter *t_i_* = *T/N_i_* (*t_1_*, *t_2_* and *t_h_* for populations *P_1_*, *P_2_* and *H*, respectively). This method, already applied to the Neolithic Transition in previous works [8], [68], [69], is described in Chikhi *et al.* [33] and implemented in LEA [70] and ParLEA [71]. It has been shown that both the cultural and demic diffusion models can be seen as extreme cases of an admixture model, whereby two or more parental populations mixed in the past to produce the hybrid ancestors of present-day populations [1], [8]. Thus, in extreme cases of admixture, with no genetic contribution of one of the parental populations, we would expect that the gene pool of present-day populations is similar to the Mesolithic HGs, in the case of CDM, or to the Neolithic farmers, in the case of DDM.

### aDNA and Coalescent Analysis

We used Bayesian Serial SimCoal software [72], [73] to simulate data, by tracing the ancestry of the female modern samples and incorporating aDNA samples of both *HG* and farmers, for each of the three models described in Figure 2. We explored 2,500 parameter combinations using fifty equally spaced values, sampled from the priors for both N_UP_ (ranging from 10 to 5,000) and N_N_ (between 1,000 and 100,000), that is using the same range as in [27].

The selection of the best demographic model was carried out under an ABC framework [32], [37]. The same approach was applied to estimate parameters for the selected model (SDG) [31], [32]. The validation of this procedure is fully described in SI Material Methods (see also table S3).

Further details regarding the admixture analysis in modern and ancient data and the data sets used in this study may be found in *Text S1*.

## Supporting Information

Figure S1 **Admixture model used by the Chikhi** ***et al.*** [**1**] **method.** See SI Methods for more details and reference information.
(TIF)

Figure S2 **Palaeolithic contribution to modern European (** ***p_1_*** **) posterior distributions, for each of the European populations analysed, using NRY data** [**2**] **.** Each curve corresponds to the analysis of a specific hybrid (admixed) population. In **(A)** are represented all the populations used in this study and in **(B)** are the populations used as negative control. See Text S1 for more details and reference information.
(TIF)

Figure S3 **Linear regression of Neolithic contribution (1−** ***p_1_*** **), against geographical distance from the Near East, using NRY data** [**2**] **.** In **(A)** are represented all the populations used in this study and in **(B)** are the populations used as negative control. Mean values for each population are represented by red circles. See SI Methods for more details and reference information.
(TIF)

Figure S4 **Linear regression of Neolithic contribution (1−** ***p_1_*** **) against geographical distance from the Near East, using NRY data** [**2**] **.** In **(A)** are represented the Caucasus populations (note the different scale on the x-axis) and in **(B)** are the European Islands population samples (Cyprus, Sardinian, UK and Ireland) used in this study. Mean values for each population are represented by red circles. See Text S1 for more details and reference information.
(TIF)

Figure S5 **Distributions of the** ***t_i_*** **’s for all populations, using NRY** [**2**] **.** **(A)** Posterior distributions of *t_1_*. The different curves represent the amount of genetic drift, since the admixture event, between the present sample of Basques and the ancestral populations of HG that interbred with the incoming farmers. **(B)** Posterior distributions of *t_2_*. As in **(A)**, but for the drift between the Near East and the first farmer populations. The colour codes are as in Figure S2A. See Text S1 for more details and reference information.
(TIF)

Figure S6 **Distributions of the** ***t_i_*** **’s for all populations, using mtDNA** [**3**] **(A) Posterior distributions of** ***t_1_*** **. (B) Posterior distributions of** ***t_2_*** **(see Figure S5 for a more detailed explanation).** Note that the panel B has a different scale on the x-axis compared to panel A and Figure S5. See Text S1 for more details and reference information.
(TIF)

Figure S7 **Estimated effective population sizes for the admixed populations (** ***N_h_*** **) and their distance from the Near East.** The *N_h_* values were calculated for the Rosser *et al.* [2] dataset using archaeological dates from table S4 and a generation time of 25 years. See Text S1 for more details and reference information.
(TIF)

Figure S8 **Genetic diversity and differentiation, across Europe.** In **(A)**, the *H_e_* values for each European population analysed are regressed against the geographic distance from the Near East, both for NRY (solid circles) and mtDNA (open circles). The linear regressions calculated from these points are represented by the solid (NRY) and dashed (mtDNA) lines. In **(B),** each point represents pairwise *F_ST_* values, between European populations and the Near East, regressed against distance from the latter. The symbol and line codes are as in (A).
(TIF)

Figure S9 **Split with differential growth model (SDG), with name of the demes.**
(TIF)

Table S1 **Demographic parameters estimated under the Split with Differential Growth (SDG) model.** Weighted (**ω**) median, 5% and 95% percentiles values are represented for the *N_e_* at the Neolithic and Upper Palaeolithic. Deme 1 and 2 correspond to the demes without and with differential growth, respectively (see Figure S9).
(PDF)

Table S2 **Maximum probability of obtaining genetic differentiation (** ***F_ST_*** **) values larger than those observed in the real data.** Maximum probability values of obtaining a simulated F_ST_ value higher than that observed (P_s>o_), for each of the models (TP - Total Panmixia, S - Split, SDG - Split with Differential Growth) and pairwise comparisons analysed (see Figure 3). See Text S1 for more details and reference information.
(PDF)

Table S3 **Validation of the ABC model selection procedure.** Each row corresponds to the percentage of times that a model (TP - Total Panmixia, S - Split, SDG - Split with Differential Growth) was assigned to each of the models, by a higher posterior probability. When data are simulated under the S model our results show that a significant proportion of the data sets are identified as being generated under another model (and as many as 44.7% are assigned to the TP model). This is less the case for the data generated under the TP model (but still they represent as much as 25% altogether) and even less under the SDG model. Thus despite non negligible error rates, these simulations suggest that there is a bias favouring the TP model, and much less the S and SDG models. One reason for this is that the ABC algorithm used here followed the procedure of Bramanti and colleagues [5], and was only based on three statistics, which were available. However, the results also show that the SDG model is the model which is most easily identified with nearly 88% of positive results. Given that the results obtained from the real data provide no support for the TPM, and less than 5% for the S model, we are confident that the inference of the model is unlikely to be incorrect hence demonstrating the importance of differential growth. This explains why Haak *et al*. [6] were unable to explain the observed *F_ST_* values with their split model. See Text S1 for more details and reference information.
(PDF)

Table S4 **Calibrated radiocarbon dates of Neolithic archaeological sites (from Pinhasi** ***et al.*** [**4**] **.** Location and type of Neolithic culture (EN- Early Neolithic, LBK- Linear Pottery Culture) are also represented in this table. See Text S1 for reference information.
(PDF)

Text S1 **Support Materials and Methods and references to SI Tables and SI Figures**.
(PDF)
