# Supplementary figures and images for: Female and Male Perspectives on the Neolithic Transition in Europe: Clues from Ancient and Modern Genetic Data
Source: PLoS One. 2013 Apr 17;8(4):e60944. doi: 10.1371/journal.pone.0060944 (PMC3629215; doi:10.1371/journal.pone.0060944)

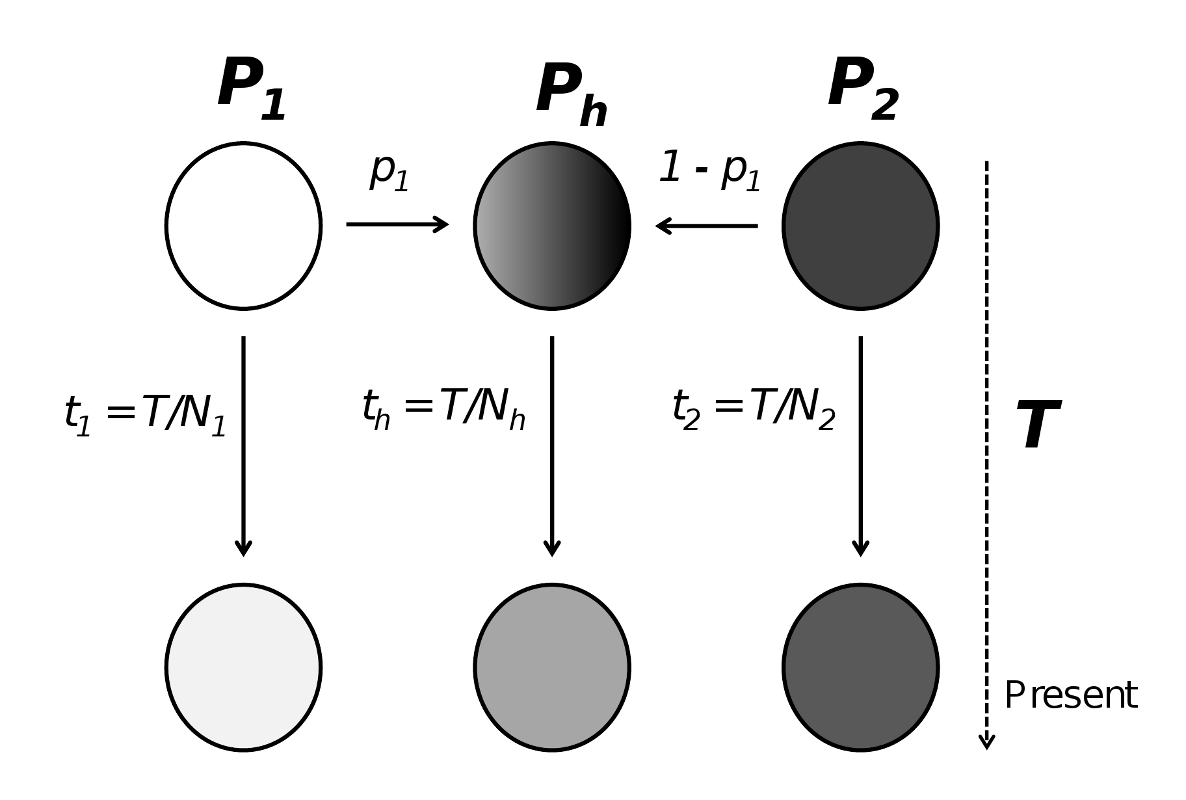

Supplement: Figure S1 — Admixture model used by the Chikhi et al. [1] method. See SI Methods for more details and reference information. (TIF) [file pone.0060944.s001.tif]

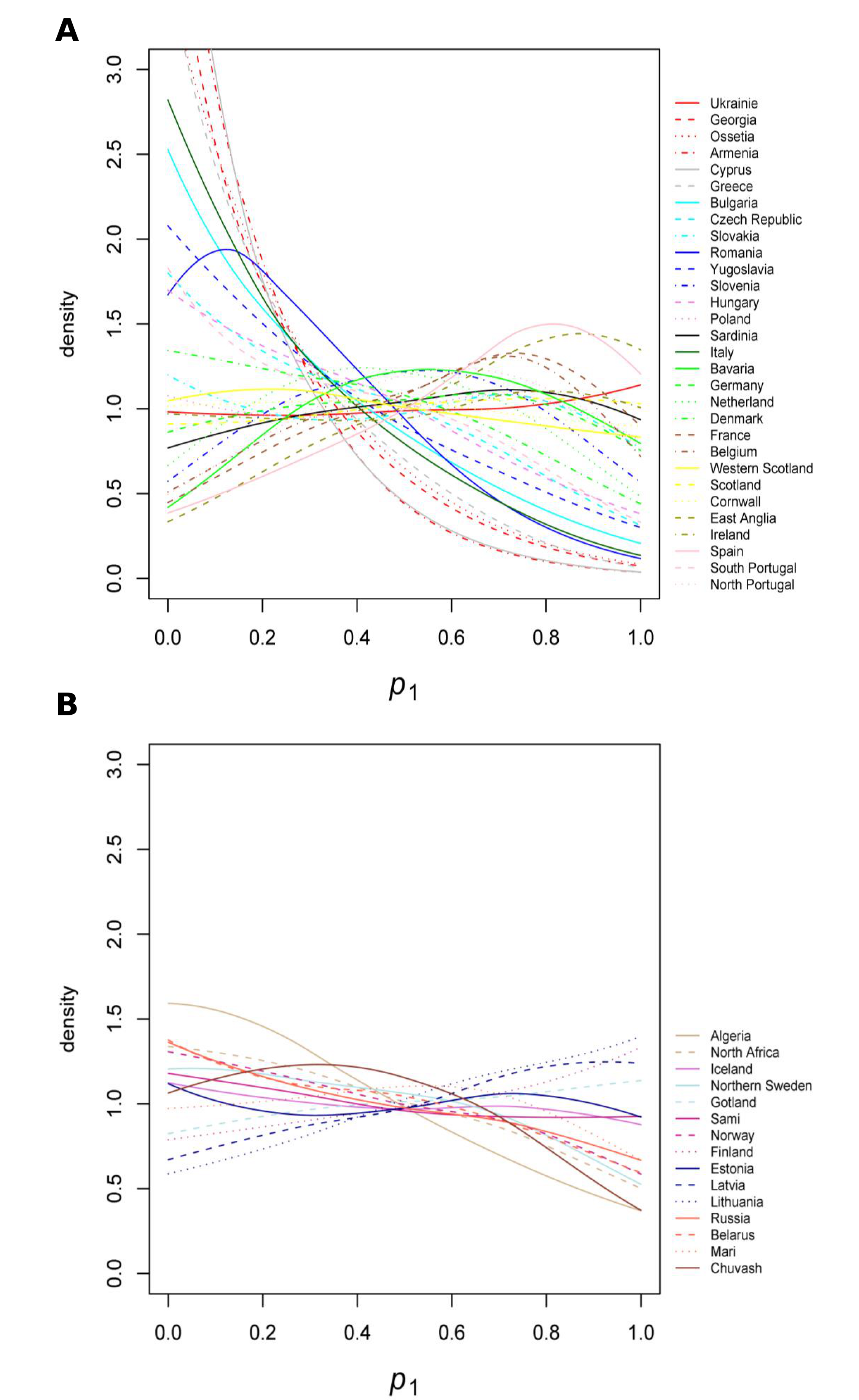

Supplement: Figure S2 — Palaeolithic contribution to modern European ( p1 ) posterior distributions, for each of the European populations analysed, using NRY data [2] . Each curve corresponds to the analysis of a specific hybrid (admixed) population. In (A) are represented all the populations used in this study and in (B) are the populations used as negative control. See Text S1 for more details and reference information. (TIF) [file pone.0060944.s002.tif]

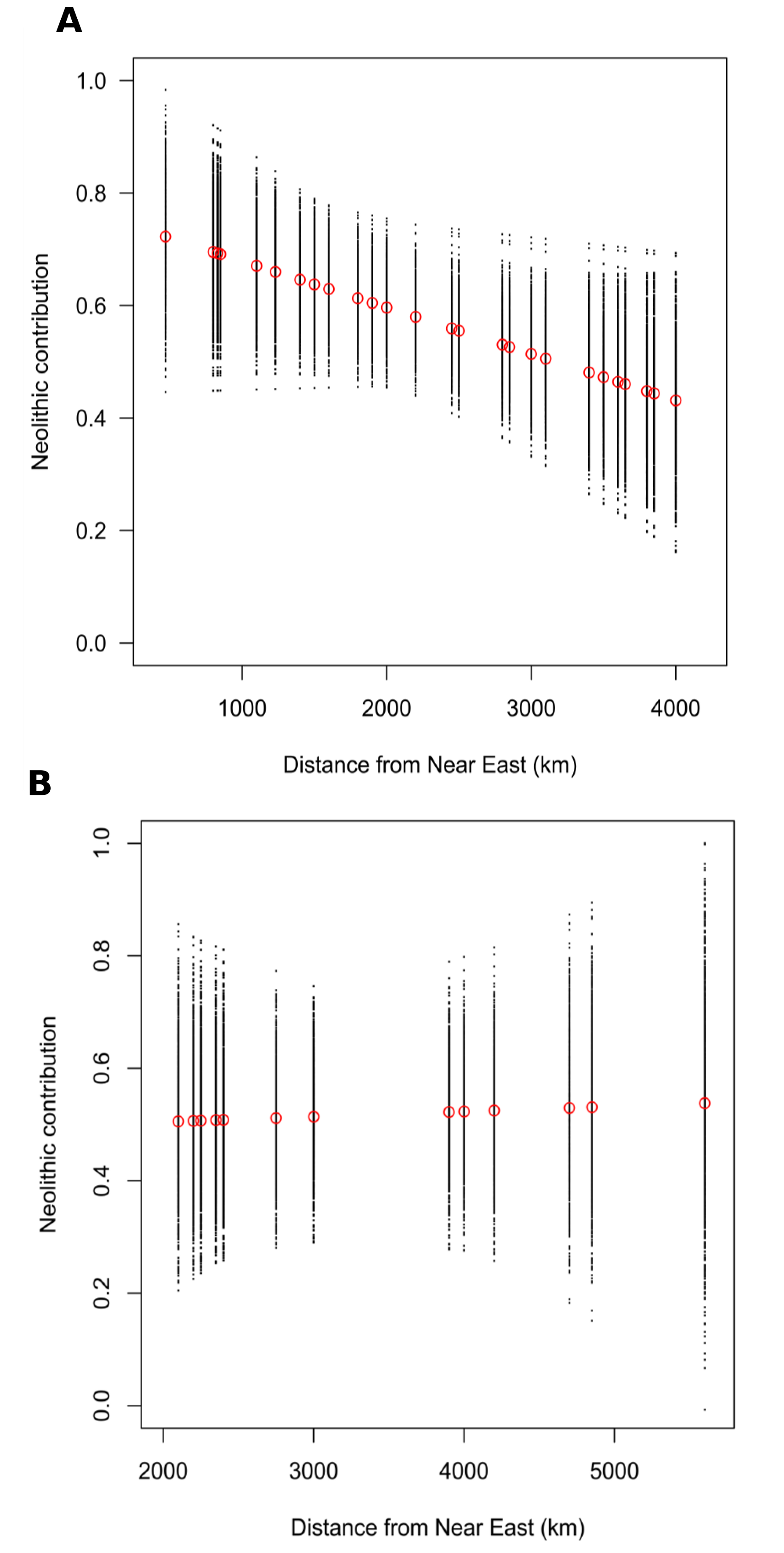

Supplement: Figure S3 — Linear regression of Neolithic contribution (1− p1 ), against geographical distance from the Near East, using NRY data [2] . In (A) are represented all the populations used in this study and in (B) are the populations used as negative control. Mean values for each population are represented by red circles. See SI Methods for more details and reference information. (TIF) [file pone.0060944.s003.tif]

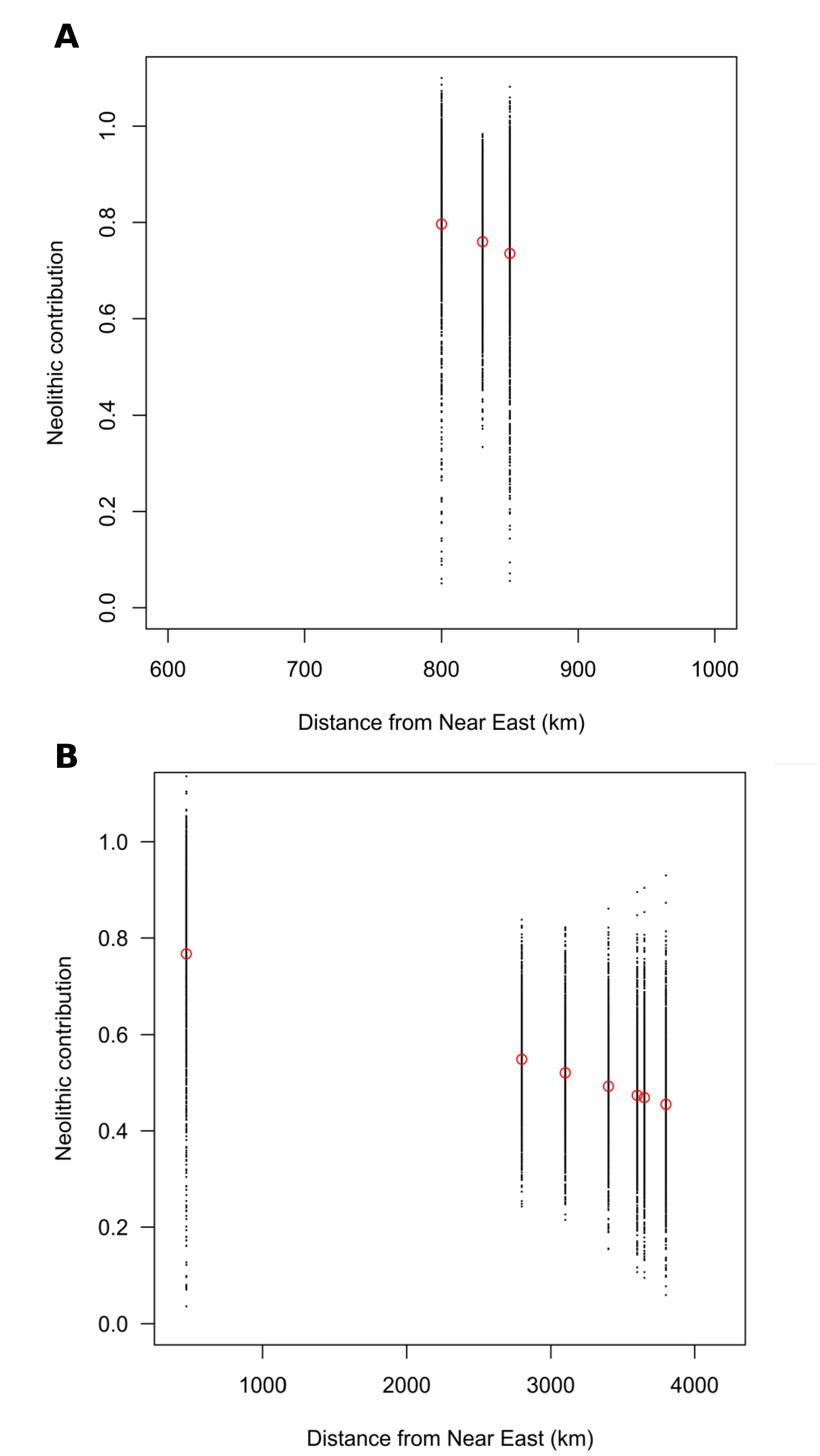

Supplement: Figure S4 — Linear regression of Neolithic contribution (1− p1 ) against geographical distance from the Near East, using NRY data [2] . In (A) are represented the Caucasus populations (note the different scale on the x-axis) and in (B) are the European Islands population samples (Cyprus, Sardinian, UK and Ireland) used in this study. Mean values for each population are represented by red circles. See Text S1 for more details and reference information. (TIF) [file pone.0060944.s004.tif]

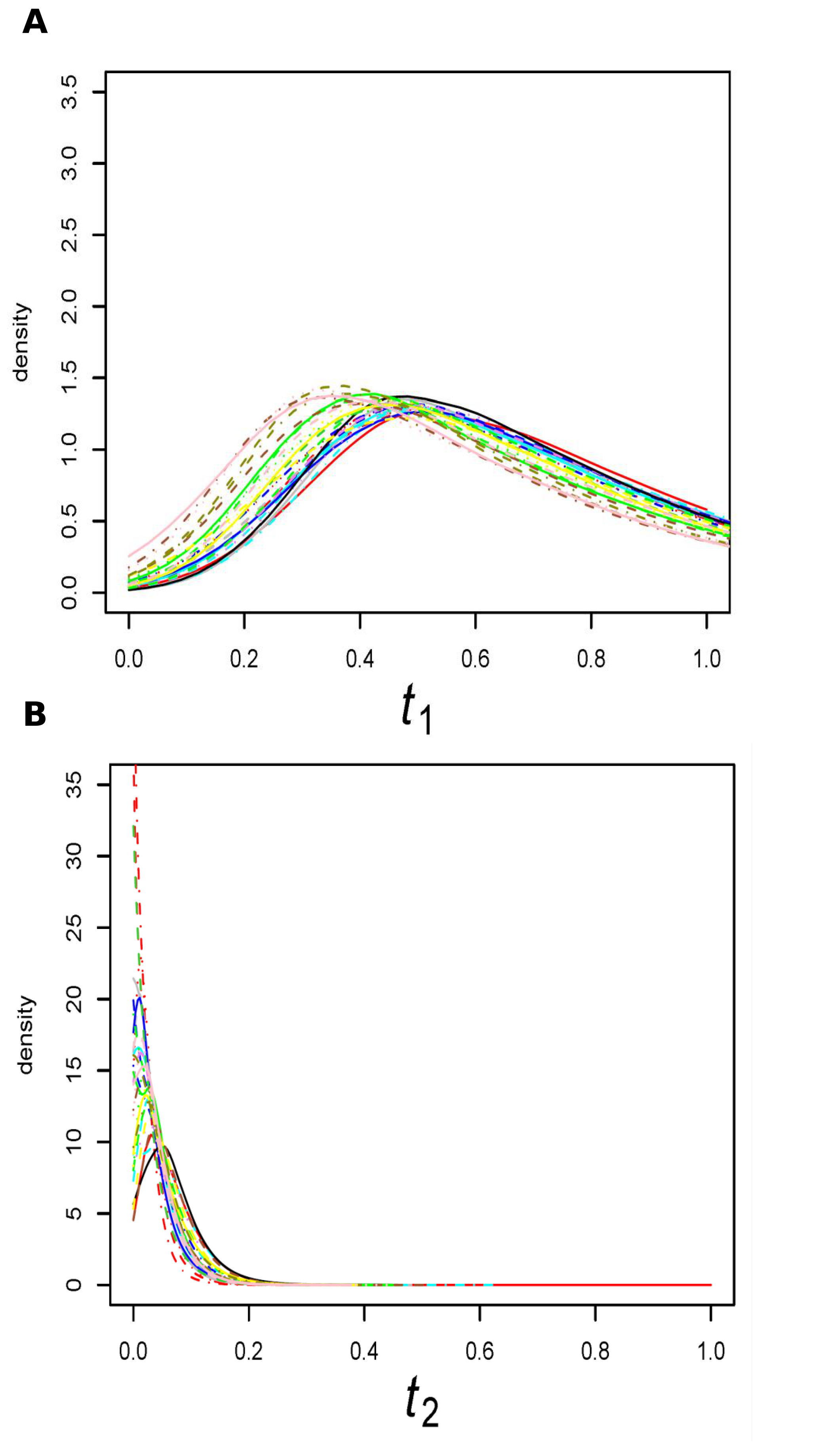

Supplement: Figure S5 — Distributions of the ti ’s for all populations, using NRY [2] . (A) Posterior distributions of t1. The different curves represent the amount of genetic drift, since the admixture event, between the present sample of Basques and the ancestral populations of HG that interbred with the incoming farmers. (B) Posterior distributions of t2. As in (A), but for the drift between the Near East and the first farmer populations. The colour codes are as in Figure S2A. See Text S1 for more details and reference information. (TIF) [file pone.0060944.s005.tif]

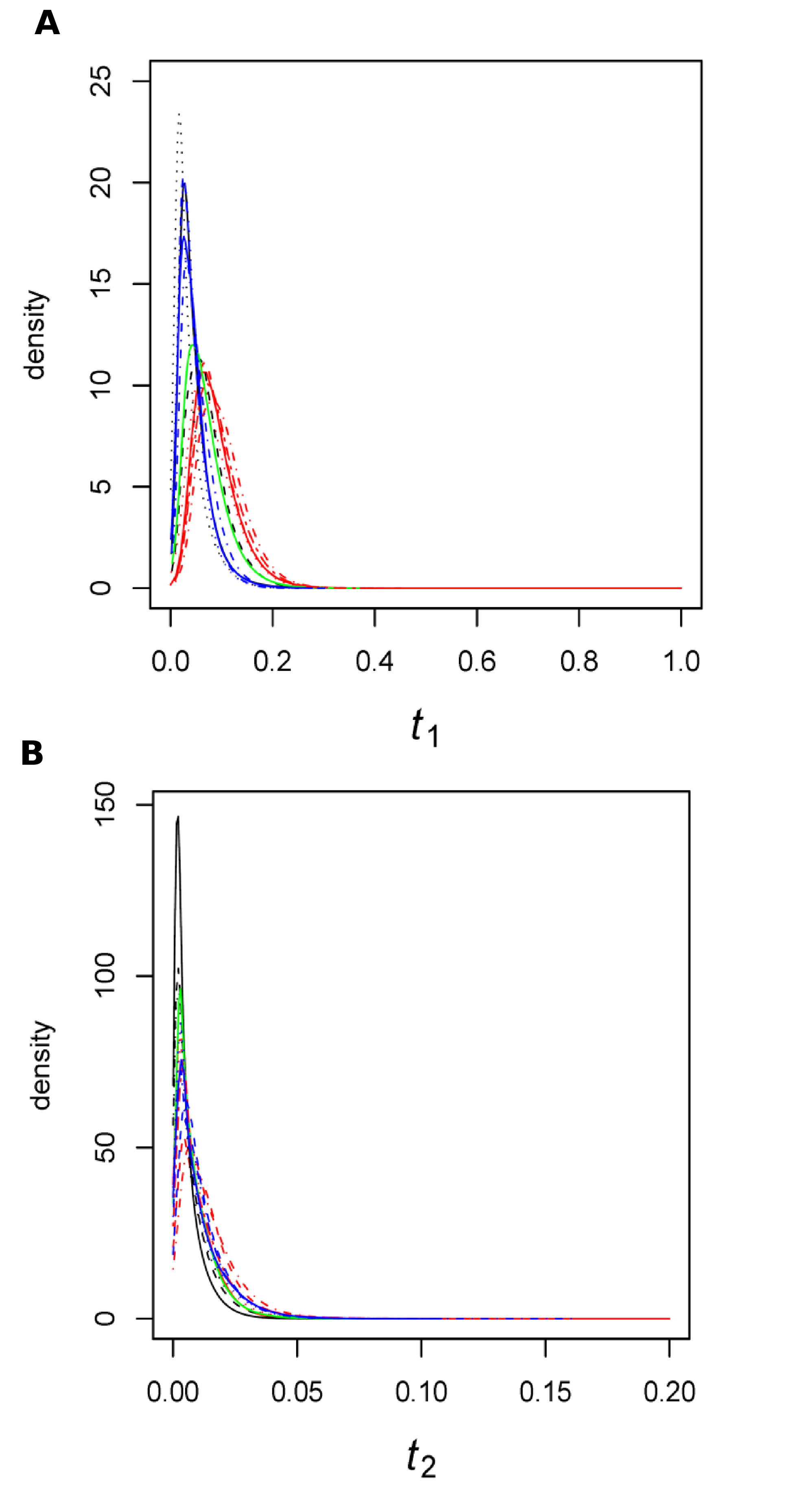

Supplement: Figure S6 — Distributions of the ti ’s for all populations, using mtDNA [3] (A) Posterior distributions of t1 . (B) Posterior distributions of t2 (see Figure S5 for a more detailed explanation). Note that the panel B has a different scale on the x-axis compared to panel A and Figure S5. See Text S1 for more details and reference information. (TIF) [file pone.0060944.s006.tif]

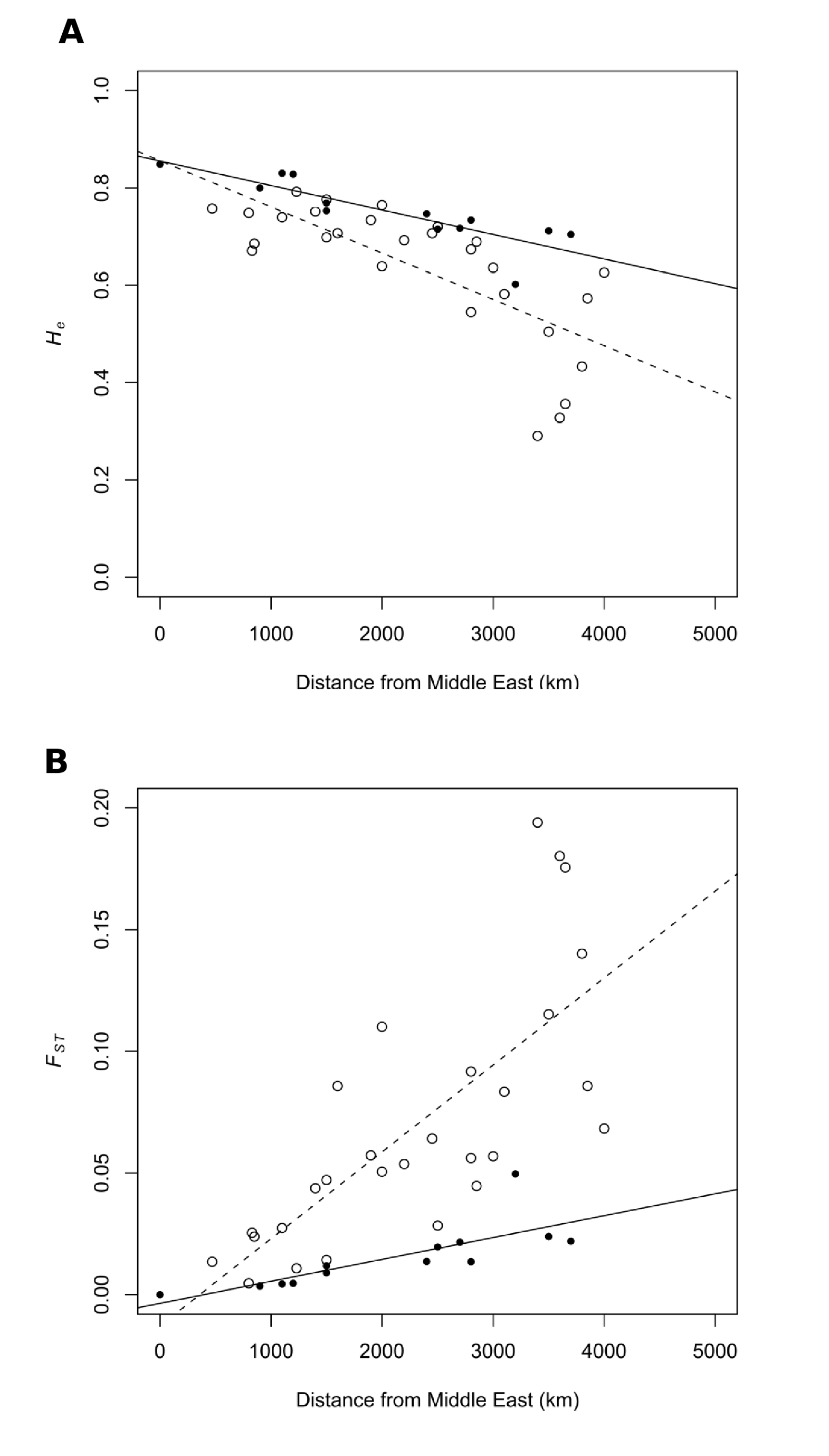

Supplement: Figure S8 — Genetic diversity and differentiation, across Europe. In (A), the He values for each European population analysed are regressed against the geographic distance from the Near East, both for NRY (solid circles) and mtDNA (open circles). The linear regressions calculated from these points are represented by the solid (NRY) and dashed (mtDNA) lines. In (B), each point represents pairwise FST values, between European populations and the Near East, regressed against distance from the latter. The symbol and line codes are as in (A). (TIF) [file pone.0060944.s008.tif]

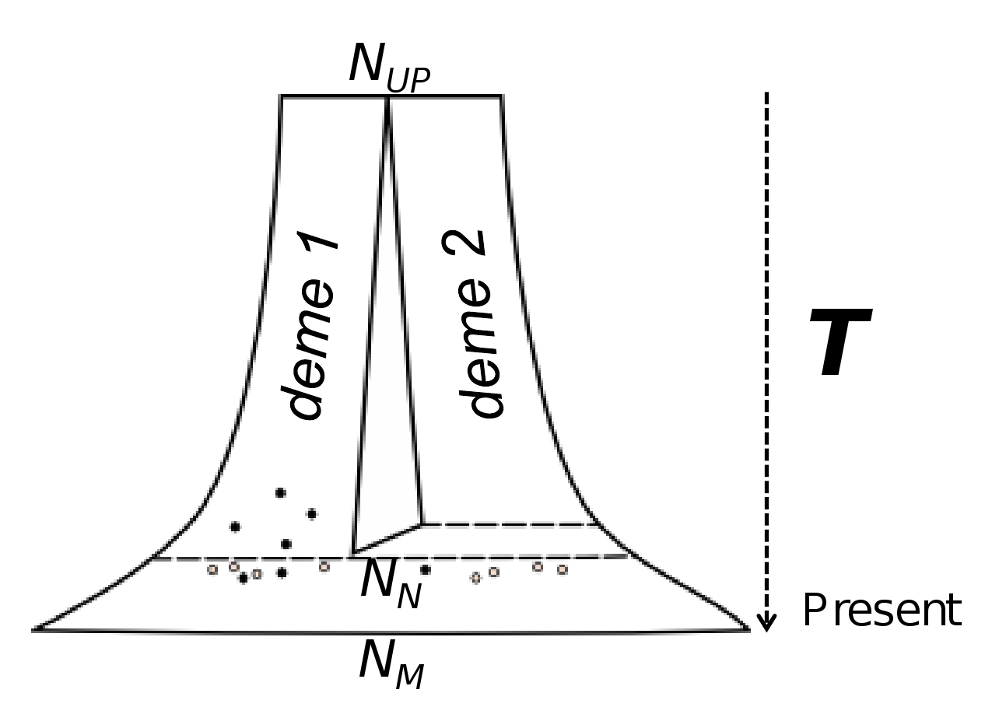

Supplement: Figure S9 — Split with differential growth model (SDG), with name of the demes. (TIF) [file pone.0060944.s009.tif]
