# Supplementary material for: Female and Male Perspectives on the Neolithic Transition in Europe: Clues from Ancient and Modern Genetic Data
Source: PLoS One. 2013 Apr 17;8(4):e60944. doi: 10.1371/journal.pone.0060944 (PMC3629215; doi:10.1371/journal.pone.0060944)
Supplement: Table S1 — Demographic parameters estimated under the Split with Differential Growth (SDG) model. Weighted (ω) median, 5% and 95% percentiles values are represented for the Ne at the Neolithic and Upper Palaeolithic. Deme 1 and 2 correspond to the demes without and with differential growth, respectively (see Figure S9). (PDF) [file pone.0060944.s010.pdf]

Table S1. Demographic parameters estimated under the Split with Differential Growth (SDG) model.

		**ω Median**	**ω 5% Perc.**	**ω 95% Perc.**	**Prior**
**N_e Neolithic**	Total	18,374.80	3,529.00	77,274.60	U: 1,000-100,000
	Deme 1	967.10	185.74	4,067.09	
	Deme 2	17,407.70	3,343.26	73,207.51	
**N_e Upper Palaeolithic**	Total	2,248.12	297.64	4,717.56	U: 10-5,000
	Deme 1	1,124.06	148.82	2,358.78	
	Deme 2	1,124.06	148.82	2,358.78	

Weighted (ω) median, 5% and 95% percentiles values are represented for the N_e at the Neolithic and Upper Palaeolithic. Deme 1 and 2 correspond to the demes without and with differential growth, respectively (see Figure S9).
