# Supplementary material for: Female and Male Perspectives on the Neolithic Transition in Europe: Clues from Ancient and Modern Genetic Data
Source: PLoS One. 2013 Apr 17;8(4):e60944. doi: 10.1371/journal.pone.0060944 (PMC3629215; doi:10.1371/journal.pone.0060944)
Supplement: Table S2 — Maximum probability of obtaining genetic differentiation ( FST ) values larger than those observed in the real data. Maximum probability values of obtaining a simulated FST value higher than that observed (Ps>o), for each of the models (TP - Total Panmixia, S - Split, SDG - Split with Differential Growth) and pairwise comparisons analysed (see Figure 3). See Text S1 for more details and reference information. (PDF) [file pone.0060944.s011.pdf]

Table S2. Maximum probability of obtaining genetic differentiation (F_{ST}) values larger than those observed in the real data.

**Models**	$\mathbf{P_{s > o}}$		
	HG vs. Farmers	*HG vs.* Modern	Farmer *vs.* Modern
TP (this study)	0.018	0.032	0.152
TP (Bramanti *et al.* [5])	0.022	0.028	-
S	0.132	0.278	0.192
SDG	0.990	1.000	0.612

Maximum probability values of obtaining a simulated F_{ST} value higher than that observed ($P_{s>o}$), for each of the models (TP - Total Panmixia, S - Split, SDG - Split with Differential Growth) and pairwise comparisons analysed (see Figure 3). See Text S1 for more details and reference information.
