# Supplementary material for: Female and Male Perspectives on the Neolithic Transition in Europe: Clues from Ancient and Modern Genetic Data
Source: PLoS One. 2013 Apr 17;8(4):e60944. doi: 10.1371/journal.pone.0060944 (PMC3629215; doi:10.1371/journal.pone.0060944)
Supplement: Table S3 — Validation of the ABC model selection procedure. Each row corresponds to the percentage of times that a model (TP - Total Panmixia, S - Split, SDG - Split with Differential Growth) was assigned to each of the models, by a higher posterior probability. When data are simulated under the S model our results show that a significant proportion of the data sets are identified as being generated under another model (and as many as 44.7% are assigned to the TP model). This is less the case for the data generated under the TP model (but still they represent as much as 25% altogether) and even less under the SDG model. Thus despite non negligible error rates, these simulations suggest that there is a bias favouring the TP model, and much less the S and SDG models. One reason for this is that the ABC algorithm used here followed the procedure of Bramanti and colleagues [5], and was only based on three statistics, which were available. However, the results also show that the SDG model is the model which is most easily identified with nearly 88% of positive results. Given that the results obtained from the real data provide no support for the TPM, and less than 5% for the S model, we are confident that the inference of the model is unlikely to be incorrect hence demonstrating the importance of differential growth. This explains why Haak et al. [6] were unable to explain the observed FST values with their split model. See Text S1 for more details and reference information. (PDF) [file pone.0060944.s012.pdf]

Table S3. Validation of the ABC model selection procedure.

	**% Attribution**		
	**TP**	**S**	**SDG**
**TP**	74.7	21.3	4.0
**S**	44.7	38.1	17.2
**SDG**	2.6	9.6	87.8

Each row corresponds to the percentage of times that a model (TP - Total Panmixia, S - Split, SDG - Split with Differential Growth) was assigned to each of the models, by a higher posterior probability. When data are simulated under the S model our results show that a significant proportion of the data sets are identified as being generated under another model (and as many as 44.7% are assigned to the TP model). This is less the case for the data generated under the TP model (but still they represent as much as 25% altogether) and even less under the SDG model. Thus despite non negligible error rates, these simulations suggest that there is a bias favouring the TP model, and much less the S and SDG models. One reason for this is that the ABC algorithm used here followed the procedure of Bramanti and colleagues [5], and was only based on three statistics, which were available. However, the results also show that the SDG model is the model which is most easily identified with nearly 88% of positive results. Given that the results obtained from the real data provide no support for the TPM, and less than 5% for the S model, we are confident that the inference of the model is unlikely to be incorrect hence demonstrating the importance of differential growth. This explains why Haak *et al*. [6] were unable to explain the observed F_{ST} values with their split model. See Text S1 for more details and reference information.
