# Supplementary material for: Female and Male Perspectives on the Neolithic Transition in Europe: Clues from Ancient and Modern Genetic Data
Source: PLoS One. 2013 Apr 17;8(4):e60944. doi: 10.1371/journal.pone.0060944 (PMC3629215; doi:10.1371/journal.pone.0060944)
Supplement: Table S4 — Calibrated radiocarbon dates of Neolithic archaeological sites (from Pinhasi et al. [4] . Location and type of Neolithic culture (EN- Early Neolithic, LBK- Linear Pottery Culture) are also represented in this table. See Text S1 for reference information. (PDF) [file pone.0060944.s013.pdf]

Table S4. Calibrated radiocarbon dates of Neolithic archaeological sites (from Pinhasi *et al.* [4]).

Location	Archaeological site	Culture	Dates (Yrs cal BP)
Georgia	Arkb1	Pottery Neolithic	7937
Cyprus	Cypro-EPPNB	Kissonega-Mylouthkia	10494
Greece	Knossos	EN	8946
Bulgaria	Polyanista-Platoto1	EN	8145
Czech Republic	Bylany	LBK	7604
Slovakia	Sturovo	LBK	7146
Romania	Trestiana	Starcevo,Cri	7539
Yugoslavia	Apatin	Starcevo	7932
Hungary	Endrod	Köros	7765
Poland	Strezelce	LBK	7150
Italy	Praia di Mare	EN	8324
Germany	Klein Denkte	LBK	8803
Netherlands	Geleen	LBK	7317
Denmark	Christiansholm Mose	Neolithic	6139
France	Pontcharaud	Epicardial	7930
Belgium	Omal	LBK	7412
Scotland	Boghead Mound	Neolithic	6839
Cornwall	Carn Bea	Neolithic	5761
East Anglia	Strawberry Hill	Neolithic	7677
Ireland	Carrowmore	Neolithic	6038
Spain	Cueva del Nasciemento	EN	7637
Portugal	Pena D'Água	Cardial	7629
Northern Sweden	Skoteholm	Neolithic	6297
Lithuania	Daktariske	Neolithic	6317

Location and type of Neolithic culture (EN- Early Neolithic, LBK- Linear Pottery Culture) are also represented in this table. See Text S1 for reference information.
