# Supplementary material for: Female and Male Perspectives on the Neolithic Transition in Europe: Clues from Ancient and Modern Genetic Data
Source: PLoS One. 2013 Apr 17;8(4):e60944. doi: 10.1371/journal.pone.0060944 (PMC3629215; doi:10.1371/journal.pone.0060944)
Supplement: Text S1 — Support Materials and Methods and references to SI Tables and SI Figures. (PDF) [file pone.0060944.s014.pdf]

SUPPORTING INFORMATION METHODS

FEMALE AND MALE PERSPECTIVES ON THE NEOLITHIC TRANSITION IN EUROPE: CLUES FROM ANCIENT AND MODERN GENETIC DATA

Rita Rasteiro (rr147@le.ac.uk)

Instituto Gulbenkian de Ciência, Rua da Quinta Grande, Oeiras, Portugal

Present address: Department of Genetics/School of Historical Studies, University of Leicester, Leicester, UK

Lounès Chikhi (chikhi@igc.gulbenkian.pt; lounes.chikhi@univ-tlse.fr)

Instituto Gulbenkian de Ciência, Rua da Quinta Grande, Oeiras, Portugal

CNRS, Université Paul Sabatier, ENFA, UMR 5174 EDB (Laboratoire Evolution et Diversité Biologique), Toulouse, France

Université de Toulouse; UPS; EDB (Laboratoire Evolution et Diversité Biologique), Toulouse, France

CONTENTS

1. Estimating admixture between hunter-gatherers and farmers ... 2

1.1. The admixture model ... 2

1.2. Populations used ... 3

1.3. Choice of Parental Populations ... 3

1.4. Validation of the admixture analysis with negative controls ... 4

1.5. Regression Analysis ... 4

1.6. F_{ST} analysis ... 5

2. Analysis of ancient DNA (aDNA) ... 5

2.1. Populations' datasets ... 5

2.2. Demographic models: testing for the continuity and discontinuity hypotheses ... 5

2.3. Distribution of pairwise F_{ST} values across models and validation of our simulation approach ... 7

2.4. Approximate Bayesian Computations (ABC) for model selection and parameter estimation ... 8

References to SI Methods ... 9

References to SI figures and SI tables ... 11

1. ESTIMATING ADMIXTURE BETWEEN HUNTER-GATHERERS AND FARMERS

1.1. *THE ADMIXTURE MODEL*

We applied a Bayesian full-likelihood method, described in Chikhi *et al.* [1], to make statistical inference on the Neolithic Transition. The original method is implemented in the LEA software [2], including a recent parallelized version of it [3] and has been applied to the Neolithic Transition in several regions of the world [4-6]. However, it may be worth emphasizing that the idea of using admixture models to study the Neolithic transition is implicit in several previous population genetic studies (*e.g.* [7]) and it has been shown that both the cultural (CDM) and demic (DDM) diffusion models can be seen as extreme cases of an admixture model, whereby two or more parental populations mixed in the past to produce the hybrid ancestors of present-day populations [5,8]. Thus, in extreme cases of admixture, with no genetic contribution of one of the parental populations, we would expect that the gene pool of present-day populations is similar to the Mesolithic HGs, in the case of CDM, or to the Neolithic farmers, in the case of DDM.

The method used here makes the admixture model very explicit (**Figure S1**) and assumes that T generations in the past, an ''admixed'' population H (representing the European populations), is formed by members of two independent parental populations, P_1 (representing the local hunter-gatherers, per instance) and P_2 (representing the incoming farmers), whose contributions to H are p_1 and p_2 ($p_2 = 1 - p_1$), respectively. After the admixture event, the three populations are assumed to evolve independently under pure genetic drift (*i.e.* mutations after admixture are assumed to be negligible). Therefore, all populations are allowed to have changed in allele frequency since the time of admixture by genetic drift. Changes in allele frequency will depend on both T and on the effective population sizes (N_1, N_2 and N_h). Genetic drift is thus modelled by the three parameters, namely $t_1 = T/N_1$, (drift in the hunter-gatherers (*HG*) since admixture), $t_2 = T/N_2$ (drift in the Near-Eastern population) and $t_h = T/N_h$ (drift in the admixed population, namely the different European populations analysed). Although very simple, by separating the effects of admixture from drift, the model should be able to capture the essential features of European prehistory as has been shown by simulation [5,9]. Note also that each analysis of a European population is performed independently with the same parental populations. This means that the method can in principle explain the genetic data in different European populations by varying any of the model parameters. We expect that if the model captures important aspects

of the Neolithic transition, the parameters that will vary most are p_1 (the admixture parameter) as a function of geographic distance from the Near East and t_h, (drift in the admixed population) as a function of both geographical distance and local effective sizes. On the contrary, for data for which an admixture model is unlikely to be meaningful, the same data set could in principle be explained by increasing or decreasing drift in any of the parental populations (t_1, t_2). This is not what we observe (see below for the validation and in the main text for the use of negative controls).

As noted above, the admixture method is implemented in the LEA software [2], which uses a MCMC algorithm to sample the posterior distributions of the model parameters (p_1, t_1, t_2 and t_h), using the full information from haplotypes frequencies observed today. For each analysis, LEA was run for 300,000 steps, as it has been shown that it is enough to reach equilibrium for single-locus data [1,3,5,9].

1.2. POPULATIONS USED

In order to compare the demographic history of both female and male lineages, we selected a large number of modern European and circum-European populations, for which haplogroup frequencies were published for both paternally- and maternally- inherited markers. The Rosser *et al.* [10] dataset comprises 3616 NRY (nonrecombining region of the Y-chromosome), for a total of 47 populations. The Richards *et al.* [11] dataset consists of 4095 individuals typed for their mitochondrial DNA (mtDNA). These data were also compared to the previously analysed NRY data of Semino *et al.* [12] to determine whether similar trends were observed across the two NRY data. Semino *et al.* [12] typed more genetic markers (and identified more haplotypes), but for a smaller sample size (n = 1007).

1.3. CHOICE OF PARENTAL POPULATIONS

Archaeological, linguistic and genetic studies suggest that the Neolithic transition started in the Near East and expanded in several directions, including a northwest movement towards Europe. To represent the descendants of the Near Eastern Neolithic farmers, most genetic studies (e.g., [5,13-14] have used samples from Turkey, Iraq, Iran, Lebanon, or Syria (i.e. the regions where farming most probably originated). We therefore used the Turkish sample for the Rosser *et al.* [10] dataset, whereas for the Richards *et al.* [11] dataset we pooled the Iraq, Syria, Palestine, Druze, Turkey and Kurds samples. To represent the descendants of the

Palaeolithic hunter-gathering populations we used the Basque population. We note that under the CDM (cultural diffusion model), all European populations are supposed to be mostly derived from local Palaeolithic ancestors, and could thus be used to that aim. However, based on linguistic and genetic evidence the Basques appear to represent one of the European populations less influenced by the Neolithic transition [15-18]. We also note that since all European population must have had some level of admixture, our approach should provide underestimates of the Near Eastern farmers in Europe.

1.4. *VALIDATION OF* THE *ADMIXTURE ANALYSIS WITH NEGATIVE CONTROLS*

Several European or circum-European populations, for which mtDNA and NRY data are available, are unlikely, due to their geographical location, to have been involved in the simple expansion and admixture model implicit in the DDM. This was the case of Iceland, Scandinavian countries like Sweden (including the Island of Gotland) and Norway, Baltic countries (Latvia and Lithuania), some Slavic samples (Russia and Belarus) and of the Uralic (Sami, Mari, Estonian and Finnish) and Altaic (Chuvash) language families. These populations were used as negative controls. Indeed, our prediction is that for these populations, the decrease of admixture proportion with increasing geographical distance from the Near East should not hold, or should be much less obvious. We also note that for some populations from the Afroasiatic language family (Algeria and the North Africa sample) the predictions are more difficult to make. We analyse these populations here, to determine whether their admixture level may provide some hint regarding the expansion of the Afro-Asiatic language, but the limited number of samples makes this a conjecture that will need more samples to be tested.

1.5. *REGRESSION ANALYSIS*

A linear regression approach was used to detect, quantify, and assess the significance of any geographical trend in admixture proportions across Europe [5]. Based on the samples available for the genetic analyses, the geographic distance was calculated from the middle point: i) of Turkey [10] and ii) between Syria and Turkey [11]. Given that we do not have access to the exact value of p_1 for the samples analysed, but rather to a posterior distribution which presents some level of uncertainty, the regression was performed by repeatedly sampling from the p_1 distributions in the following manner. For each of the European samples, one p_1 value was randomly sampled from the corresponding posterior distribution. A linear regression was then calculated

between this set of values and geographic distance. This process was repeated 1,000 times to obtain the empirical distribution of regression curves. A similar approach was used for T/N_h.

1.6. F_{ST} ANALYSIS

To further analyse the genetic structure of the populations, and to ascertain the differences between male and female variation patterns we used F_{ST} statistics, computed according to Nei [19], as it only requires allele frequencies. The pairwise F_{ST} values were calculated for both NRY and mtDNA datasets, using the Near Eastern samples against all the other populations. These values were then plotted against the geographical distance from the same locations used for the regression analyses.

2. ANALYSIS OF ANCIENT DNA (ADNA)

2.1. POPULATIONS' DATASETS

At the time of writing and analysis, the two largest European aDNA data sets available were those of Haak *et al.* [20] and of Bramanti *et al.* [21]. Both present mtDNA data from Central European *HG* [21] and from early LBK/AVK (Linear Pottery/Alföld Linear Pottery) farmers [20] skeletons, respectively. They were analysed with modern mtDNA data from the same geographical regions following the original authors [21].

2.2. DEMOGRAPHIC MODELS: TESTING FOR THE CONTINUITY AND DISCONTINUITY HYPOTHESES

The aDNA used in the present study were taken from two studies (see above) which reached opposite conclusions regarding the continuity *versus* discontinuity hypothesis in Europe. The study of Haak *et al.* [20] claimed that the change in haplotype frequency between Neolithic and modern samples could not be explained by drift alone, particularly due to the high frequency of the N1a haplotype, which was found at a frequency of 25% in the aDNA samples and is nearly absent in present-day European populations. They thus suggested that the Neolithic farmers were not the ancestors of modern-day Europeans and favoured a continuity hypothesis. Bramanti and colleagues [21] used a simple panmictic model to ask whether there was continuity between local Central European *HG* aDNA samples and modern-day samples from the same geographical region. They also used aDNA samples from Neolithic farmers, and concluded that the continuity hypothesis should be

rejected, i.e. that present-day Europeans are not descendants from the local Palaeolithic populations. One serious problem with this study is that it assumes total panmixia and hence cannot actually test for genetic continuity or discontinuity. We show that this model makes unrealistic and self-contradictory assumptions. Their model assumes total panmixia across all Central Europe, across all human populations (*i.e.* farmers and *HG* are assumed to be part of the same panmictic population) over the whole period of Europe colonization (45,000 years). Such extreme assumption, as we show, explains why they rarely observed the high F_{ST} values that are computed from real data. We show that by using very simple structured models, the high F_{ST} values observed in real data are actually easily generated.

To do this we performed coalescent simulations under three different sets of models. First, we simulated data under the model of Bramanti and colleagues [21] to validate our approach and reproduce their results. We named this model Total Panmixia (TP) for the reasons explained above. The TP model **(Figure 2A)** assumes that *HG* and farmers are part of the same panmictic population over Central Europe and were never separated into different populations or communities. The Bramanti model also assumes a single modern female effective population size N_M (12,000,000) and two periods of exponential growth: i) the first starting with an Upper Palaeolithic (UP) population of effective size N_{UP}, sampled from an ancestral African female population of constant size 5,000, corresponding to the initial colonization of Central Europe 45,000 years ago and ii) the second following the Neolithic Transition 7,500 years ago, from a population of effective size N_N. Both N_{UP} and N_N population sizes were allowed to vary between 10 to 5,000 and 1,000 to 100,000, respectively [21]. To avoid making the rather strong assumption of panmixia between *HG* and farmers communities, while keeping the models simple and allowing comparisons with their results, we built two models that are similar but allow for some population structure. In the Split Model (S) **(Figure 2B)** we assumed that the Upper Palaeolithic population was structured in two sub-populations of equal size, 45,000 years ago. These sub-populations were assumed to grow independently (no gene flow) until they joined at the beginning of the Neolithic, in Central Europe. For simplicity and to avoid having some Palaeolithic samples in one of the two subpopulations and others in the other subpopulation we assumed that all the Palaeolithic sequences were sampled from the same subpopulation, as shown in the **Figure 2B**. The main reason for using this model is that it is probably the simplest structured model imaginable under the framework proposed by Bramanti *et al.* [21]. It corresponds, for instance, to a scenario where *HG* where subdivided into two main populations (one in Central Europe, and

the other following a southern route) that joined during the Neolithic, with no genetic contribution from other populations. We also used a more complex splitting model that we named the Split with Differential Growth (SDG) model **(Figure 2C)**. The SDG model is similar to the S model but one of the two sub-populations was allowed to have a higher growth rate between 10,000 and 7,500 years ago. It is compatible with a scenario where the "left" population corresponds to *HG*, whereas the other one corresponds to Near Eastern farmers arriving and mixing with *HG* during the Neolithic expansion. This kind of model is an admixture model [5,14]. It is important to note, that for technical reasons, in the SDG model, we constrained one of the subpopulations (deme 1, corresponding to the HG) at the Neolithic to have a size 1/20th of N_N. (see **Figure S9**) [8].

Note that all models allowed the same parameters to vary, including the growth rates which were computed on the basis of population size values which in turn were sampled from the priors.

2.3. DISTRIBUTION OF PAIRWISE F_{ST} VALUES ACROSS MODELS AND VALIDATION OF OUR SIMULATION APPROACH

We used Bayesian Serial SimCoal software (BayeSSC) [22-23] to simulate aDNA and modern DNA data, by tracing the ancestry of the female modern samples and incorporating ancient DNA samples of both *HG* and farmers. We used the same parameter values (and/or priors) for sequence sizes, mutations rates, transition bias, distribution of mutations rate among sites, populations effective sizes and periods of time as in [21].

We explored 2,500 parameter combinations using fifty equally spaced values sampled from the priors for both N_{UP} (ranging from 10 to 5,000) and N_N (between 1,000 and 100,000), as in [21]. For each pairwise combination we performed 500 independent coalescent simulations, hence corresponding to a total of 3,750,000 simulations (1,250,000 simulations for each of the three models). Three sets of sequences were sampled from the coalescent simulations according to the sizes of the observed sequence data (*HG*, farmers and modern Central Europeans) and their corresponding ages. We then computed the pairwise F_{ST} values in the simulated data and compared them to the values observed in the real data. The proportion of times where the simulated F_{ST} was greater than the observed F_{ST} was recorded, for each combination of N_{UP} and N_N values as in [21] (see **Figure 3**). We also computed whether the observed F_{ST} values were within the 95% credible interval for each parameter combination. Scripts were written in the R language [24] to create the infiles read

by BayeSSC, to analyse the results and to produce the plots in **Figure 3**. The 2,500 points forming the grid and for which the probabilities were estimated, were used to produce the interpolated plots with the *filled.contour* R function [24]. The observed pairwise F_{ST} values found by Bramanti and colleagues [21] and used in this study are: 0.163 for *HG vs.* farmers, 0.0858 for *HG vs.* moderns and 0.058 for farmers *vs.* moderns.

2.4. *Approximate Bayesian Computations (ABC) for model selection and parameter estimation*

In order to determine which of the three demographic models explained best the data and then estimate the demographic parameters of interest we used an ABC approach [25-26]. We performed 1,500,000 simulations for each model (4,500,000 simulations in total) and selected the 1% simulations that best explained the observed data (this was also done using the 0.1% best-fitting simulations and provided the same results). Following Bramanti *et al.* [21], and to facilitate comparison between studies, we used the three pairwise F_{ST} values used by these authors between the *HG*, farmers and modern samples as summary statistics. The simulations were performed with the BayeSCC program, but contrary to the previous section we did not use a grid of values but rather proper *a priori* distributions. The ABC inference procedure was performed using the *abc* R package [27]. The *postpr* function was used to select the best model (estimate the posterior probability of each of the three models). This was done using two approaches (i) the Beaumont *et al.* [25] multinomial logistic regression (MLR) model, and (ii) the nonlinear conditional heteroscedastic (NCH) model that uses a neural network approach [1]. The latter approach uses a non-linear regression correction to minimize departure from non-linearity, that enhances accuracy when compared to the regression algorithm proposed by Beaumont *et al.* [26-29]. For the model that was selected we then we estimated the selected model's parameters of interest (N_{UP} and N_N), using the 1% simulations (15,000 values) associated with the shortest Euclidian distances from the observed data. The NCH regression-ABC method, proposed by [1], jointly with a logit transformation, was used to estimate the parameters based on the observed and simulated pairwise F_{ST} values.

The model selection approach was validated by calculating the power to recover the true model. For that, we took randomly 1,000 datasets, from the original BayeSSC runs for ABC analysis, for each of the three demographic models. We thus assigned each of these datasets to a model, by using again the function *postpr*.

However, this time we used the pairwise F_{ST} values of the simulated datasets as pseudo-observed summary statistics. Finally, we counted the number of times that the true model was correctly identified.

REFERENCES TO SI METHODS

1. Chikhi L, Bruford MW, Beaumont MA (2001) Estimation of admixture proportions: a likelihood-based approach using Markov chain Monte Carlo. Genetics 158: 1347-1362.
2. Langella O, Chikhi L, Beaumont MA (2001) LEA (likelihood-based estimation of admixture): a program to estimate simultaneously admixture and time since the admixture event. Mol Ecol Notes 1: 357-358.
3. Giovannini A, Zanghirati G, Beaumont MA, Chikhi L, Barbujani G (2009) A novel parallel approach to the likelihood-based estimation of admixture in population genetics. Bioinformatics 25: 1440-1441.
4. Belle EMS, Landry P, Barbujani G (2006) Origins and evolution of the Europeans' genome: evidence from multiple microsatellite loci. Proc R Soc B 273: 1595-1602.
5. Chikhi L, Nichols RA, Barbujani G, Beaumont MA (2002) Y genetic data support the Neolithic demic diffusion model. Proc. Natl. Acad. Sci. U. S. A. 99: 11008-11013.
6. Rasteiro R, Chikhi L (2009) Revisiting the peopling of Japan: an admixture perspective. J Hum Genet 54: 349-354.
7. Barbujani G, Sokal RR, Oden NL (1995) Indo-European origins: a computer-simulation test of five hypotheses. Am J Phys Anthropol 96: 109-132.
8. Currat M, Excoffier L (2005) The effect of the Neolithic expansion on European molecular diversity. Proc R Soc B 272: 679-688.
9. Sousa VC, Fritz M, Beaumont MA, Chikhi L (2009) Approximate Bayesian Computation without summary statistics: the case of admixture. Genetics 181: 1507-1519.
10. Rosser ZH, Zerjal T, Hurles ME, Adojaan M, Alavantic D et al. (2000) Y-chromosomal diversity in Europe is clinal and influenced primarily by geography, rather than by language. Am J Hum Genet 67: 1526-1543.
11. Richards M, Macaulay V, Hickey E, Vega E, Sykes B et al. (2000) Tracing European founder lineages in the Near Eastern mtDNA pool. Am. J. Hum. Genet. 67: 1251-1276.
12. Semino O, Passarino G, Oefner PJ, Lin AA, Arbuzova S et al. (2000) The genetic legacy of Paleolithic Homo sapiens sapiens in extant Europeans: a Y chromosome perspective. Science 290: 1155-1159.
13. Barbujani G, Bertorelle G, Capitani G, Scozzari R (1995) Geographical structuring in the mtDNA of Italians. Proc Natl Acad Sci U S A 92: 9171-9175.

14. Goldstein DB, Chikhi L (2002) Human migrations and population structure: what we know and why it matters. Annu Rev Genomics Hum Genet 3: 129-152.

15. Brion M, Salas A, González-Neira A, Lareu MV, Carracedo A (2003) Insights into Iberian population origins through the construction of highly informative Y-chromosome haplotypes using biallelic markers, STRs, and the MSY1 minisatellite. Am J Phys Anthropol 122: 147-161.

16. Cavalli-Sforza LL (1998) The Basque population and ancient migrations in Europe. Munibe (Antropologia-Arqueologia) 6 (Suppl): 129-137.

17. Menozzi P, Piazza A, Cavalli-Sforza L (1978) Synthetic maps of human gene frequencies in Europeans. Science 201: 786-792.

18. Wilson JF, Weiss DA, Richards M, Thomas MG, Bradman N et al. (2001) Genetic evidence for different male and female roles during cultural transitions in the British Isles. Proc Natl Acad Sci U S A 98: 5078-5083.

19. Nei M (1977) F-statistics and analysis of gene diversity in subdivided populations. Ann Hum Genet 41: 225-233.

20. Haak W, Forster P, Bramanti B, Matsumura S, Brandt G et al. (2005) Ancient DNA from the first European farmers in 7500-year-old Neolithic sites. Science 310: 1016-1018.

21. Bramanti B, Thomas MG, Haak W, Unterlaender M, Jores P et al. (2009) Genetic Discontinuity Between Local Hunter-Gatherers and Central Europe's First Farmers. Science 326: 137-140.

22. Anderson CNK, Ramakrishnan U, Chan YL, Hadly EA (2005) Serial SimCoal: a population genetics model for data from multiple populations and points in time. Bioinformatics 21: 1733-1734.

23. Excoffier L, Novembre J, Schneider S (2000) SIMCOAL: a general coalescent program for the simulation of molecular data in interconnected populations with arbitrary demography. J Hered 91: 506-509.

24. Development Core Team R (2009) R: A language and environment for statistical computing.

25. Beaumont M (2008) Joint determination of topology, divergence time, and immigration in population trees. In: Matsumura S, Forster P, Renfrew C, editors. Simulation, genetics, and human prehistory. Cambridge: McDonald Institute for Archaeological Research. pp. 135-154.

26. Beaumont MA, Zhang W, Balding DJ (2002) Approximate Bayesian computation in population genetics. Genetics 162: 2025-2035.

27. Csillery K, Francois O, Blum MGB (2012) abc: an R package for Approximate Bayesian Computation (ABC). MEE 3: 475-479

28. Blum MGB, François O (2010) Non-linear regression models for Approximate Bayesian Computation. Stat Comput 20: 63-73.

29. Beaumont MA (2010) Approximate Bayesian Computation in Evolution and Ecology. Ann Rev Ecol Evol S 41: 379-406.

REFERENCES TO SI FIGURES AND SI TABLES

1. Chikhi L, Bruford MW, Beaumont MA (2001) Estimation of admixture proportions: a likelihood-based approach using Markov chain Monte Carlo. Genetics 158: 1347-1362.
2. Rosser ZH, Zerjal T, Hurles ME, Adojaan M, Alavantic D et al. (2000) Y-chromosomal diversity in Europe is clinal and influenced primarily by geography, rather than by language. Am J Hum Genet 67: 1526-1543.
3. Richards M, Macaulay V, Hickey E, Vega E, Sykes B et al. (2000) Tracing European founder lineages in the Near Eastern mtDNA pool. Am. J. Hum. Genet. 67: 1251-1276.
4. Pinhasi R, Fort J, Ammerman AJ (2005) Tracing the origin and spread of agriculture in Europe. PLoS Biol 3: e410.
5. Bramanti B, Thomas MG, Haak W, Unterlaender M, Jores P et al. (2009) Genetic Discontinuity Between Local Hunter-Gatherers and Central Europe's First Farmers. Science 326: 137-140.
6. Haak W, Balanovsky O, Sanchez JJ, Koshel S, Zaporozhchenko V et al. (2010) Ancient DNA from European Early Neolithic Farmers Reveals Their Near Eastern Affinities. PLoS Biol 8: e1000536.
